# Genome-Wide Analyses of Radioresistance-Associated miRNA Expression Profile in Nasopharyngeal Carcinoma Using Next Generation Deep Sequencing

**DOI:** 10.1371/journal.pone.0084486

**Published:** 2013-12-19

**Authors:** Guo Li, Yuanzheng Qiu, Zhongwu Su, Shuling Ren, Chao Liu, Yongquan Tian, Yong Liu

**Affiliations:** 1 Department of Otolaryngology Head and Neck Surgery, Xiangya Hospital, Central South University, Hunan, China; 2 Otolaryngology Major Disease Research Key Laboratory of Hunan Province, Changsha, Hunan, China; University of Barcelona, Spain

## Abstract

**Background:**

Rapidly growing evidence suggests that microRNAs (miRNAs) are involved in a wide range of cancer malignant behaviours including radioresistance. Therefore, the present study was designed to investigate miRNA expression patterns associated with radioresistance in NPC.

**Methods:**

The differential expression profiles of miRNAs and mRNAs associated with NPC radioresistance were constructed. The predicted target mRNAs of miRNAs and their enriched signaling pathways were analyzed via biological informatical algorithms. Finally, partial miRNAs and pathways-correlated target mRNAs were validated in two NPC radioreisitant cell models.

**Results:**

50 known and 9 novel miRNAs with significant difference were identified, and their target mRNAs were narrowed down to 53 nasopharyngeal-/NPC-specific mRNAs. Subsequent KEGG analyses demonstrated that the 53 mRNAs were enriched in 37 signaling pathways. Further qRT-PCR assays confirmed 3 down-regulated miRNAs (miR-324-3p, miR-93-3p and miR-4501), 3 up-regulated miRNAs (miR-371a-5p, miR-34c-5p and miR-1323) and 2 novel miRNAs. Additionally, corresponding alterations of pathways-correlated target mRNAs were observed including 5 up-regulated mRNAs (*ICAM1*, *WNT2B*, *MYC*, *HLA-F* and *TGF-β1*) and 3 down-regulated mRNAs (*CDH1*, *PTENP1* and *HSP90AA1*).

**Conclusions:**

Our study provides an overview of miRNA expression profile and the interactions between miRNA and their target mRNAs, which will deepen our understanding of the important roles of miRNAs in NPC radioresistance.

## Introduction

Nasopharyngeal carcinoma (NPC) is an Epstein-Barr virus (EBV) associated cancer, which has a remarkably racial and geographic distribution and is prevalent in the Southern regions of China and Southeast Asia [1]. The incidence of NPC is up to 20 per 100 000 persons and radiotherapy is the most common treatment strategy [2]. Despite recent advances in radiation techniques, rapidly emerging evidence indicates that NPC recurrence and metastasis still account for the majority of NPC-related deaths, even after aggressive therapy [3]. Radioresistance accounts for the majority of treatment failures and leads to relapse and metastasis in NPC patients after radiotherapy. Thus, it is imperative to identify the molecular biomarkers involved in radioresistance and explore the biological processes underlying the development of radioresistance. Previous attempts to identify radioresistance-associated protein and mRNA biomarkers using genomic microarrays and proteomics have increased our knowledge of radioresistance [4,5]. However, the molecular mechanisms underlying radioresistance remain uncharacterized. Therefore, it is urgent to interpret radioresistance from a brand-new perspective. 

The discovery of microRNAs (miRNAs) established a new paradigm of post-transcriptional gene regulation. During the past decade, miRNAs have been closely linked to human diseases, including cancer. miRNAs are single stranded, endogenous, 19-25 nucleotide (nt), non-coding RNA molecules that negatively regulate target gene expression at the post-transcriptional level via binding to the 3’- or 5’-untranslated regions (UTR), leading to the degradation of target mRNAs and/or suppression of protein translation [6,7]. Human miRNA genes are frequently located at fragile sites or cancer-associated genomic regions, indicating their important roles in carcinogenesis [8,9]. Emerging evidence indicates that miRNAs are implicated in a wide variety of cellular processes, including proliferation, apoptosis, autophagy, migration, invasion and metastasis [10-12]. Therefore, miRNA targeting therapy is proposed to be a novel interfering strategy for cancer management. Numerous efforts have been made to discover and identify various miRNAs that are associated with different malignant behaviors in recent years. Interestingly, several miRNAs, including miRNA-324-3p [13], miRNA-302 [14], miRNA-31 [15], miRNA-205 [16] and let7 [17] have been recently discovered to participate in the acquisition of cancer cell radioresistance.

Initially, traditional miRNA array approaches were widely applied for miRNA identification and contributed greatly to miRNA discovery in NPC research [16,18]. However, traditional methods fail to identify novel miRNAs that are not included in array platforms, which often contain a limited number of previously-identified miRNAs. A new approach, using next generation high-throughput deep sequencing technology and massive parallel analyses of miRNAs that are widely expressed in the genome (miRNome), provides a rapid and high throughput tool that can be used to explore the large miRNA pool, and possesses obvious advantages for the identification of miRNA sequence variations and the discovery of novel miRNAs. Therefore, the present study was designed to elucidate the global miRNA expression profile that is associated with NPC radioresistance using a high-throughput deep sequencing technique.

## Materials and Methods

### Establishment of radioresistant NPC cells

Two poorly differentiated NPC cell lines, CNE-2 and 6-10B, were purchased from the Cell Center of Central South University, Changsha, China. Gradually increasing doses of irradiation were administered to NPC CNE-2 and 6-10B cells to screen for and establish the CNE-2 and 6-10B cells with enhanced radioresistant capacity (abbreviated as CNE-2-Rs and 6-10B-Rs), as previously described [13]. The cells were propagated in RPMI medium 1640 (Hyclone, Logan, UT, USA) containing 10% FBS (Gibco BRL, Gaithersburg, MD, USA) and 1% antibiotics (Gibco BRL, Gaithersburg, MD, USA) and were cultured in an incubator at 37 °C with saturated humidity and 5% CO_2_. Cells in an exponentially growing state were used for all of the following experiments. Cell viability assays with Cell Counting Kit-8 (Beyotime, China), clonogenic survival assays and flow cytometric analyses were performed to verify the radioresistant capacity as we described previously [13].

### RNA sample preperation

Total RNA was extracted using TRIzol® reagent (Invitrogen, Burlington, ON, Canada), according to the manufacturer's recommended protocol. The yield and purity of the RNA was determined by measuring the absorbance (Abs) at 260 and 280 nm. RNA samples were only used when the ratio of the Abs260 nm/Abs280 nm was > 1.8. The integrity of the RNA samples was verified using a 1% agarose gel with the RNA 6000 Nano Assay Kit and Agilent 2100 Bioanalyzer. The extracted total RNA was stored at −80 °C for later use.

### Small RNA library construction and sequencing

Approximately 10 μg of total RNA were used for sequencing with the HiSeq 2000 (Illumina, San Diego, USA) by following the manufacturer’s recommended protocols, as previously described by Mao W et al. [19]. In brief, the sRNA fractions, with lengths of 18–30 nt, were isolated using 15% denaturing polyacrylamide gel electrophoresis. After ligating sRNAs with 5’ and 3’ adaptors, the obtained short RNAs were reverse transcribed into cDNA . The resulting small RNA libraries were then sequenced using the HiSeq 2000 in a single-end manner with the planned read length set to 49 bp. 

### mRNA library construction and Sequencing

Total RNAs from CNE-2 and CNE-2-Rs cells were used for library construction. The mRNAs were selectively enriched via rRNA depletion. These samples were fragmented and then reverse transcribed into cDNA. After ee, the resulting libraries were then sequenced on Illumina HiSeq 2000 plaform in a pair-ended manner with the planned read length set to 2X90bp.

### Identification of known and novel miRNAs

**Figure 1:**
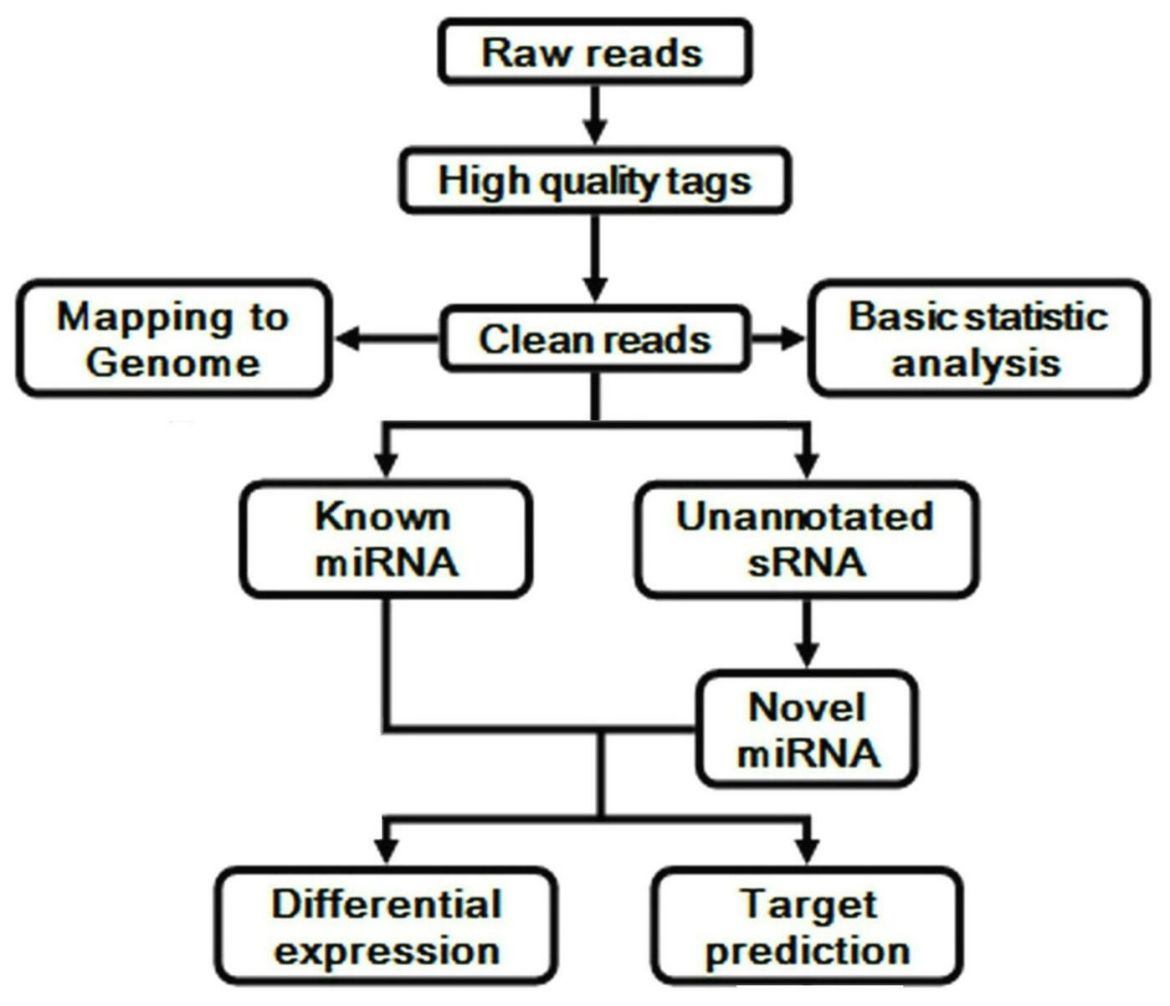
Work flow of the whole miRNA identifying procedure in details.

The raw sequences were preprocessed to filter out the low quality reads, as well as sequences without 3' primers, without insert tags, with 5' primer contaminants, with polyA tails, or with insert tags that were less than 18 nt in length. The extracted clean reads were then subjected to the miRNA database miRBase 18.0 (http://www.mirbase.org/), and a BLASTn Search was used to identify the miRNAs that were conserved in humans. The unannotated sequences were fed into MIREAP, a tool which can be used to identify both known and novel microRNAs from small RNA libraries (http://sourceforge.net/projects/mireap/). Figure 1 outlines the detailed work flow of the entire procedure. The primary data have been submitted to NCBI under BioProject accession No. PRJNA 222477.

### RNA-Seq data analyses

After preprocessing, the clean reads were mapped to reference sequences using SOAPaligner/soap2 [20], allowing for up to 5 base mismatches. To identify the genes related to radioresistance, the libraries were initially compared by pairs. The number of reads for each coding region was determined, the number of total reads was normalized between these libraries using the RPKM method (reads per kb per million reads) [21], and the log_2_ ratio of RPKM between CNE-2 and CNE-2-Rs cells was calculated. The genes that showed an absolute value of the log_2_ ratio ≥ 1 and a FDR (false discovery rate) ≤ 0.001 were considered to be potential candidates. These gene lists were then narrowed down by searching for the genes on http://www.ncbi.nlm.nih.gov/gene/ using the formula "((nasopharyngeal [All Fields] OR (nasopharyngeal [All Fields] AND carcinoma [All Fields])) OR nasopharynx [All Fields]) AND "Homo sapiens" [porgn]" to identify nasopharyngeal- and NPC-specific genes.

### Target prediction for miRNA candidates

Identification of the predicted target mRNA genes of miRNAs provides the basis for understanding the miRNA functions. Therefore, the candidate target genes of these known and novel miRNAs were analyzed using the miRNA target prediction program RNAhybrid (http://bibiserv.techfak.uni- bielefeld.de/rnahybrid/) [22]. The RefSeq mRNA sequences of the hg19 human genome (http://hgdownload.cse.ucsc.edu/goldenpath/hg19/bigZips/) were used as reference genes in the prediction. The prediction values were calculated to estimate the binding affinities of the miRNAs and their predictive target genes. The rules used for target prediction are based on those suggested by Allen et al. [23] and Schwab et al. [24].

### KEGG enrichment analyses and establishment of the microRNA gene network

KEGG is the major public pathway-related database. KEGG pathway analyses identify significantly enriched metabolic pathways or signal transduction pathways in target gene candidates by comparing them with the whole reference gene background. The formula used to calculate these similarities was previously described in literature [25]. 

### Validation of differentially expressed known and novel radioresistance- associated miRNAs in NPC using qRT-PCR

Small RNA was extracted using the miRNEasy Mini kit (Qiagen, Germantown, MD, USA). The All-in-One™ miRNA qRT-PCR Detection Kit (GeneCopoeia Inc., MD, USA) was used for the quantitative detection of mature miRNAs. Reverse transcription of miRNAs was conducted according to the manufacturer’s recommended protocol. Primers for 12 known miRNAs were purchased from (GeneCopoeia, Guangzhou, China). Primers for 2 miRNA cadidates were designed and synthesized by RiboBio Co. Real-time qRT-PCR was performed on the BIO-RAD IQTM5 Multicolor Real-Time PCR detection system (Bio-Rad). The qPCR cycle consisted of 98 °C for 2 min. and 40 cycles of 95 °C for 15 sec., followed by 62.5 °C for 40 sec. miRNA PCR quantification was conducted using the 2ΔΔCT method and was normalized to U6. The data are representative of the means of three experiments. 

### Validation of differentially expressed target mRNAs via qRT-PCR

Total RNAs were isolated from CNE-2/CNE-2-Rs and 6-10B/6-10B-Rs cells and then cDNA was synthesized from total RNA using a PrimeScript RT reagent kit with a DNA Eraser (TaKaRa, Kyoto, Japan). Primers for 12 genes were designed and synthesized (Jinsirui Biotechnology Company, Jiangsu, China, Table S1). Real-time PCR was performed on the BIO-RAD IQTM5 Multicolor Real-Time qRT-PCR detection system (Bio-Rad). The 10 ul PCR reactions included 1 ul of cDNA product and 5 ul of SYBR Premix Ex Taq II (TaKaRa). The reactions were incubated at 95 °C for 1 min, followed by 50 cycles at 95 °C for 5 sec, 60 °Cfor 60 sec, and 72 °C for 15 sec. All reactions were run in triplicate with β-Actin as internal reference. The expression levels of genes were measured conducted using the 2ΔΔCT method. 

## Results

### miRNA expression profile associated with radioresistance in NPC

**Figure 2:**
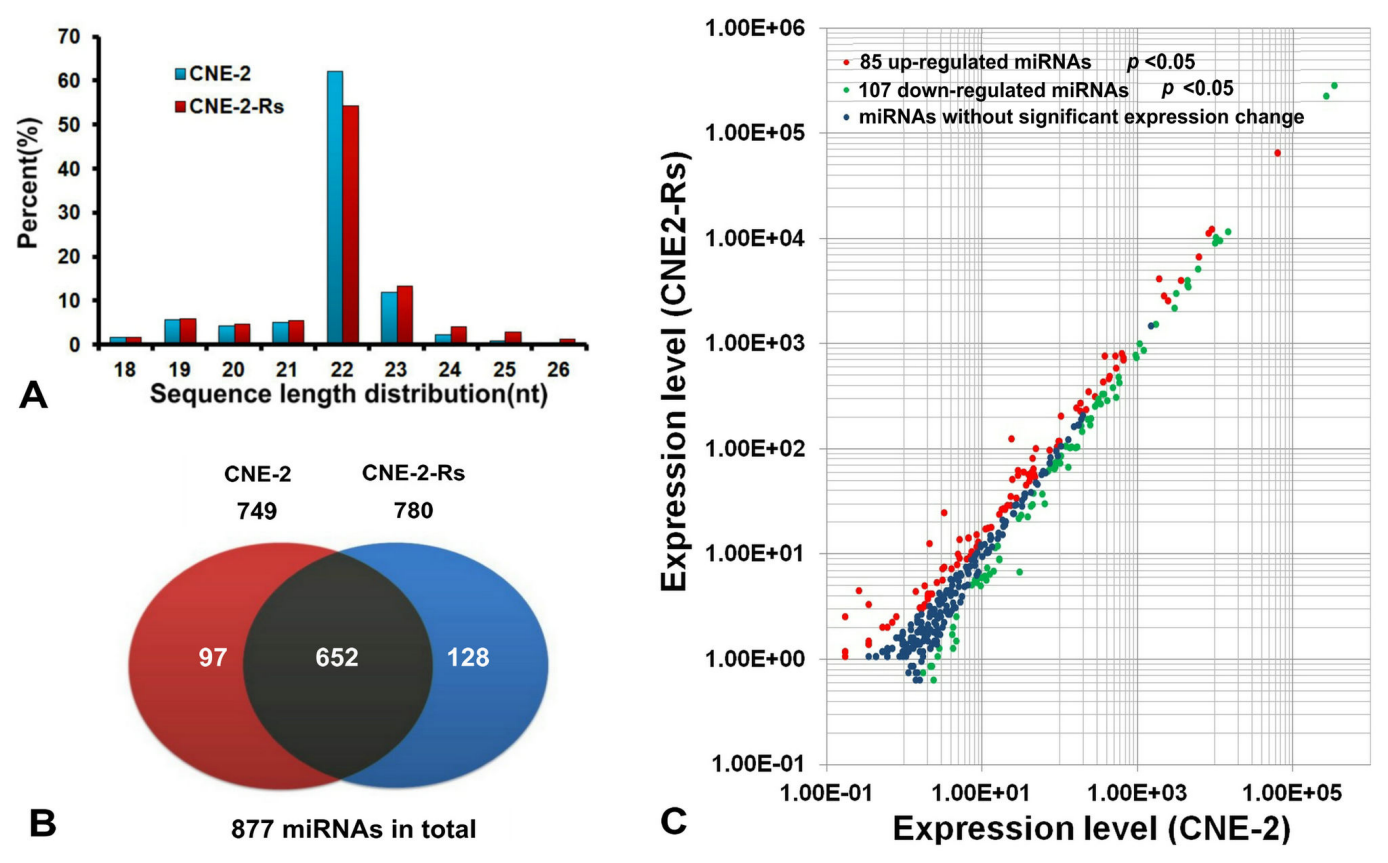
Results of the small RNA information analysis. (A) The size distribution of the small RNAs in CNE-2 and CNE-2-Rs. The sRNAs with 22 nt had the highest abundance. (B) The miRNAs identified in CNE-2 and CNE-2-Rs. The dark park were the miRNAs found in both samples, the red part and blue part showed the number of miRNAs expressed in sample CNE-2 and CNE-2-Rs, respectively. (C) Scatter plot of miR expression profiles in radiosensitive CNE-2 (x-axis) and radio-resistant CNE-2R cells (y-axis).The up-reugulated miRNAs with statistical significance were marked in red and the down-regulated ones in green (*p* < 0.05).

To identify differentially expressed miRNAs that are associated with radioresistance in NPC, small RNA libraries of radioresistant NPC cells and parental cells were sequenced using the high-throughput Illumina HiSeq 2000 system. A total of 9 989 814 and 12 078 340 raw reads were obtained from the radioresistant CNE-2-Rs cells and parental CNE-2 cells, respectively. After removing the low quality reads, 9 409 655 and 11 534 052 (CNE-2-Rs Vs. CNE-2) clean reads were left and were matched to the human genome, and 80.25% and 85.12% mapping ratios (CNE-2-Rs Vs. CNE-2) indicated original RNA samples. The length distributions of the clean reads revealed that sRNAs with 22 nt were the most abundant in both libraries (Figure 2A), which is a classic phenotype in most animals. All of the clean reads were then matched to the miRBase 18.0 database and 877 miRNAs were annotated. Among these miRNAs, 652 miRNAs were found to be expressed in both samples, while 128 and 97 miRNAs were found to only be expressed in the CNE-2-Rs and CNE-2 samples, respectively (Figure 2B). In comparison to the parental CNE-2 cells, 85 miRNAs were up-regulated and 107 miRNAs were down-regulated in the radioresistant CNE-2-Rs cells (*p* < 0.05, Figure 2C). When a fold change > 1.0 was specified, the expression of 36 up-regulated and 14 down-regulated miRNAs differed significantly (Table 1).

**Table 1:** Radioresistance associated miRNAs in NPC CNE-2 and CNE-2-Rs.

**Up-regulated miRNAs**	**Fold change**	***p* value**	**Down-regulated miRNAs**	**Fold change**	***p* value**
hsa-miR-509-3-5p	8.58	2.78E-13	hsa-miR-1246	-2.19	4.17E-38
hsa-miR-508-3p	7.50	1.11E-06	hsa-miR-324-3p	-1.87	1.62E-03
hsa-miR-371a-5p	7.41	2.48E-06	hsa-miR-4501	-1.74	4.50E-05
hsa-miR-149-3p	7.11	2.73E-05	hsa-miR-324-5p	-1.65	3.38E-05
hsa-miR-34c-5p	4.10	6.13E-12	hsa-miR-93-3p	-1.41	1.17E-02
hsa-miR-1323	3.88	4.66E-07	hsa-miR-125a-3p	-1.35	1.67E-02
hsa-miR-375	3.75	1.03E-03	hsa-miR-874	-1.34	7.76E-03
hsa-miR-504	3.25	9.11E-08	hsa-miR-185-3p	-1.29	1.15E-03
hsa-miR-139-3p	2.93	1.20E-44	hsa-miR-212-3p	-1.22	4.93E-02
hsa-miR-199a-5p	2.75	4.23E-03	hsa-miR-4454	-1.12	1.84E-02
hsa-miR-382-3p	2.75	4.23E-03	hsa-miR-1307-5p	-1.12	4.18E-31
hsa-miR-4753-5p	2.61	8.15E-03	hsa-miR-196b-3p	-1.07	4.55E-03
hsa-miR-139-5p	2.58	1.33E-20	hsa-miR-345-5p	-1.05	3.27E-07
hsa-miR-206	2.38	2.32E-175	hsa-miR-3676-3p	-1.00	1.05E-05
hsa-miR-551b-5p	2.10	5.41E-03			
hsa-miR-1291	1.99	9.64E-03			
hsa-miR-1294	1.99	9.64E-03			
hsa-miR-466	1.96	1.85E-03			
hsa-miR-4798-5p	1.73	4.12E-03			
hsa-miR-129-1-3p	1.71	1.37E-03			
hsa-miR-3126-5p	1.69	3.07E-03			
hsa-miR-215	1.65	4.13E-05			
hsa-let-7b-3p	1.46	6.19E-05			
hsa-miR-23a-5p	1.42	6.21E-11			
hsa-miR-194-5p	1.21	2.86E-05			
hsa-miR-877-5p	1.21	2.12E-05			
hsa-miR-423-5p	1.14	0.00E+00			
hsa-miR-199a-3p	1.11	4.02E-08			
hsa-miR-199b-3p	1.11	4.02E-08			
hsa-miR-92b-5p	1.10	8.39E-30			
hsa-miR-550a-5p	1.06	4.63E-03			
hsa-miR-7-5p	1.06	7.49E-24			
hsa-miR-122-5p	1.04	3.71E-43			
hsa-miR-449c-5p	1.04	1.33E-05			
hsa-miR-766-5p	1.03	1.65E-03			
hsa-miR-192-5p	1.00	6.78E-297			

### Identification of novel radioresistance-associated miRNAs in NPC

The characteristic hairpin structure of miRNA precursors can be used to predict novel miRNAs [26]. The routinely used prediction software MIREAP (BGI, China) (http://sourceforge.net/projects/mireap/) was used to predict novel miRNAs. By mapping all of the unique sRNA sequences to the human genome and predicting the hairpin structures for their flanking sequences, 32 novel miRNA candidates were obtained, 9 of which significantly differed and had fold changes > 1.0 (Fold change > 1.0, *p* < 0.05 and MFE < -20 kcal/mol, Table 2). Moreover, details regarding all of the 9 novel miRNAs were recorded in Table 2.

**Table 2:** Detailed information of novel miRNAs.

**Name**	**Length**	**Sequence**	**Location in chromosomes**	**MEF (kcal/mol)**	**Fold change**	***p*-value**
Candidate-10	21	AGGCAGCGTTTTGGATCCCTC	chr1:228768744:228768832	-32.74	-10.22	1.95E-36
Candidate-7	21	GAAGCAGCGCCTGTCGCAACT	chr17:76136813:76136903	-43.5	-1.63	1.75E-02
Candidate-17	23	TGAGTGTGTGTGTGTGAGTGTGA	chr8:79679467:79679541	-20.3	2.14	1.33E-03
Candidate-21	22	TATAAAATGGGGGTAGTAAGAC	chr11:66701904:66701979	-51	6.87	1.35E-04
Candidate-29	22	TTTGAATGTAGGAACCGATGGA	chr5:15259866:15259940	-43.4	7.22	1.23E-05
Candidate-24	20	GGCTGTGATGTTTATTAGCT	chr17:41105504:41105583	-20	7.58	5.00E-07
Candidate-20	21	TGTGTTTGTGTATGTGTATGT	chr10:57267082:57267152	-24	7.73	1.01E-07
Candidate-26	19	AGGCAGCGTTTTGGATCCCT	chr1:228768744:228768832	-32.74	8.41	6.83E-12
Candidate-30	21	TCGGGCGGGAGTGGTGGCTTT	chr6:28918820:28918902	-21.5	16.28	0.00E+00

### Construction of the miRNA-mRNA regulatory network

**Figure 3:**
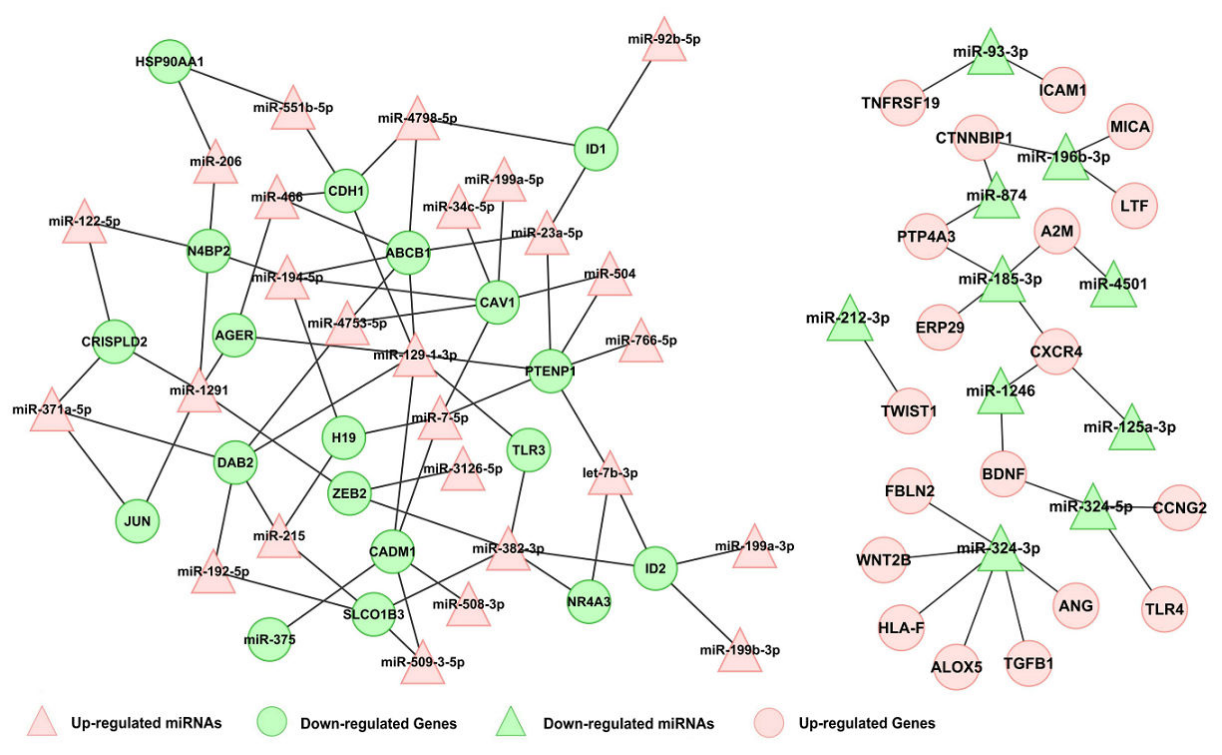
Network of the miRNAs and their prediction target gene. miRNA-gene interactions were built into a bipartite network. The red triangles indicate the up-regulated miRNAs, the blue triangles indicate the down-regulated miRNAs. The red circles indicate the up-regulated target genes, the blue circles indicate the down-regulated target genes (prediction value > 1).

miRNAs influence cancer behaviors by regulating their target mRNAs. Bioinformatic tools that were used for target gene predictions, such as miBASE [27], TargetScan [28], and PicTar [29], revealed more than 10^4^ genes in our miRNA expression profiles. Therefore, it was difficult to identify the actual target mRNAs of the miRNAs associated with NPC radioresistance in our current study. To efficiently reduce the number of target mRNAs associated with NPC radioresistance, a series of strategies were applied as follows: (1) Differentially expressed mRNAs associated with radioresistance were constructed by comparing CNE-2-Rs cells and the parental CNE-2 cells using sequencing (1214 up-regulated mRNAs and 634 down-regulated mRNAs, Table S2), (2) the target mRNA pool of the 50 miRNAs outlined above was predicted using RNAhybrid (30728 up-regulated mRNAs and 27161 down-regulated mRNAs), (3) the mRNA intersection of the two mRNA profiles outlined above was identified (453 up-regulated mRNAs and 517 down-regulated mRNAs), and (4) the mRNAs that remained after step (3) were further narrowed down by searching for these mRNAs on http://www.ncbi.nlm.nih.gov/gene/ using the formula "((nasopharyngeal [All Fields] OR (nasopharyngeal [All Fields] AND carcinoma [All Fields])) OR nasopharynx [All Fields]) AND "Homo sapiens" [porgn]". Finally, 33 up-regulated and 20 down-regulated genes and their relationships to the miRNAs are listed in Table S3. An miRNA-mRNA regulatory network was drawn in Figure 3, in which the miRNAs and target mRNAs with high binding affinities (prediction values > 1.0) were included. In the end, a limited number of radioresistant mRNAs remained, which further facilitated our investigation of the miRNA-mRNA regulatory network.

### KEGG pathway analyses of the target genes

To better understand the functions of the radioresistant miRNAs, all of the predicted target mRNAs were subjected to KEGG Pathway analyses. The results revealed that radioresistance-associated miRNAs and their predicted targets may function mainly via 37 signaling pathways (Table 3). A total of 33 up-regulated genes were found to be enriched in 22 pathways, including those related to HTLV-I infection, pathways in cancer and cell adhesion molecules (CAMs) etc, and 20 down-regulated genes were enriched in 15 pathways, including those related to pathways in cancer, the toll-like receptor and CAMs signaling pathway etc. The identification of these pathways will facilitate future mechanistic investigations of these miRNAs both *in vitro* and *in vivo*.

**Table 3:** KEGG-pathway analysis of target genes of known miRNAs.

**Pathways**			**Number of target genes**	***p*-value**
**Up-regulated target genes KEGG-Pathways**			**33**	
ko05166	HTLV-I infection		6	3.8.E-03
ko05200	Pathways in cancer		5	1.9.E-02
ko04514	Cell adhesion molecules (CAMs)		4	9.3.E-03
ko04650	Natural killer cell mediated cytotoxicity		4	9.6.E-03
ko05169	Epstein-Barr virus infection		4	1.9.E-02
ko04060	Cytokine-cytokine receptor interaction		4	1.9.E-02
ko04144	Endocytosis		4	3.1.E-02
ko05219	Bladder cancer		3	9.0.E-03
ko05144	Malaria		3	9.0.E-03
ko05323	Rheumatoid arthritis		3	1.5.E-02
ko04350	TGF-beta signaling pathway		3	1.9.E-02
ko05145	Toxoplasmosis		3	2.7.E-02
ko04310	Wnt signaling pathway		3	3.9.E-02
ko04670	Leukocyte transendothelial migration		3	4.9.E-02
ko05416	Viral myocarditis		3	4.9.E-02
ko05216	Thyroid cancer		2	1.9.E-02
ko05330	Allograft rejection		2	2.7.E-02
ko04672	Intestinal immune network for IgA production		2	2.9.E-02
ko04940	Type I diabetes mellitus		2	3.0.E-02
ko05332	Graft-versus-host disease		2	3.5.E-02
ko05320	Autoimmune thyroid disease		2	3.5.E-02
ko05210	Colorectal cancer		2	4.0.E-02
**Down-regulated target genes KEGG-Pathways**			**20**	
ko05200		Pathways in cancer	5	1.3.E-02
ko04620		Toll-like receptor signaling pathway	3	1.4.E-02
ko04514		Cell adhesion molecules (CAMs)	3	2.7.E-02
ko05168		Herpes simplex infection	3	3.7.E-02
ko05210		Colorectal cancer	2	3.7.E-02
ko05213		Endometrial cancer	2	3.7.E-02
ko05218		Melanoma	2	4.3.E-02
ko05140		Leishmaniasis	2	4.3.E-02
ko05323		Rheumatoid arthritis	2	4.3.E-02
ko05133		Pertussis	2	4.3.E-02
ko05031		Amphetamine addiction	2	4.3.E-02
ko05142		Chagas disease (American trypanosomiasis)	2	4.3.E-02
ko04350		TGF-beta signaling pathway	2	4.8.E-02
ko04662		B cell receptor signaling pathway	2	4.8.E-02
ko05215		Prostate cancer	2	4.8.E-02

### Validation of differentially expressed known and novel radioresistance- associated miRNAs in NPC

**Figure 4:**
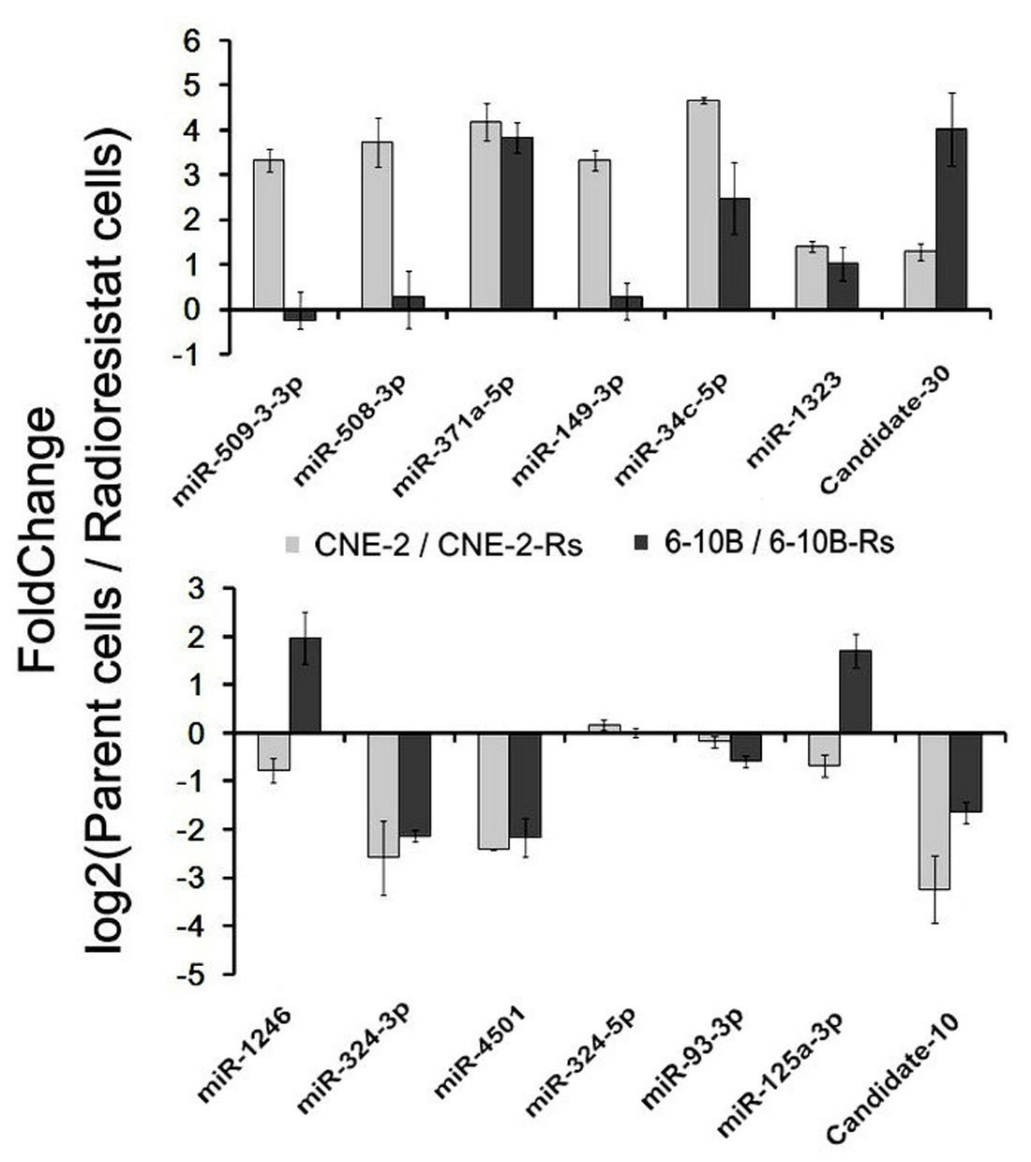
Differential miR expression between radioreisistant cell and their parent cell. Relative expression levels of several representative miRNAs with differential expression levels were presented, including 2 novel miRNAs and 10 known miRNAs.

Due to the above differential miRNA and mRNA expression profiles were derived from single CNE-2 and CNE-2-Rs cells, in order to increase the credibility of our further validation assays, another radioresistant NPC 6-10B-Rs was established and its radioresistant capability was also confirmed as our previous description [13] (Figure S1). qRT-PCR assays were then performed to confirm the top 6 up-regulated and 6 down-regulated known miRNAs and 2 novel miRNAs in radioresistant CNE-2-Rs and 6-10B-Rs cells and their parental sensitive cells. In accordance with the sequencing data, qRT-PCR results confirmed that 3 miRNAs (miR-371a-5p, miR-34c-5p, and miR-1323) were overexpressed, while 3 miRNAs (miR-324-3p, miR-93-3p, and miR-4501) were down-regulated in radioresistant NPC cells. Moreover, the expression of Candidate-30 was increased and the expression of Candidate-10 was decreased correspondingly in radioresistant NPC cells (Figure 4). However, we have to note that miR-324-5p has no significant differential expression and 5 miRNAs (miR-149-3p, miR-1246, miR-125a, miR-508-3p and miR-509-3-5p) have an inconsistent expression style between CNE-2-Rs and 6-10-Rs cells.

### Validation of predicted target mRNAs

**Figure 5:**
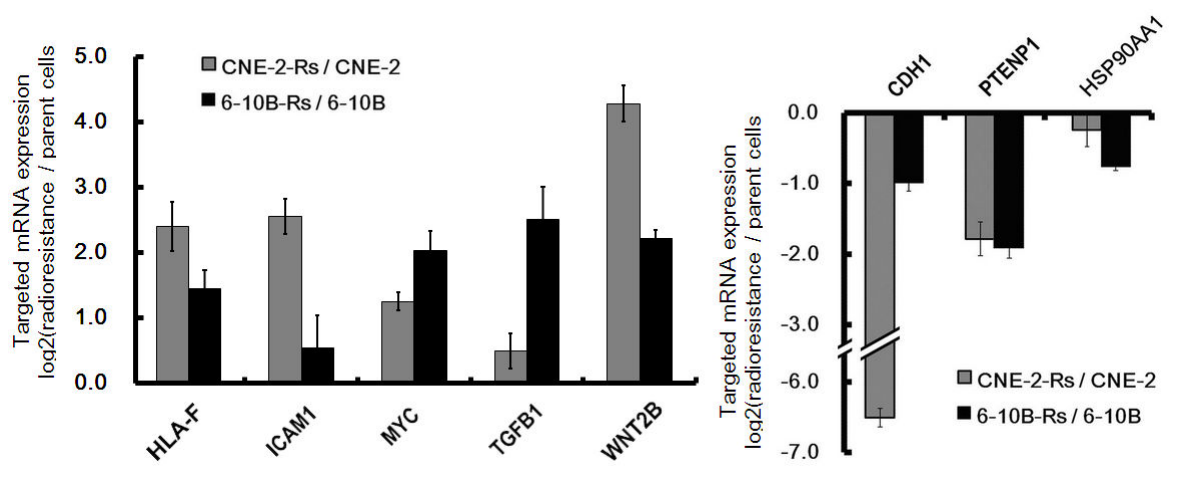
Differential mRNA expression between radioreisistant cell and their parent cell. (A) Target genes mRNAs of down-regulated miRNAs with differential expression levels were presented (*p* < 0.05). (B) Target genes mRNAs of up-regulated miRNAs with differential expression levels were presented (*p* < 0.05).

Confirmation of pathways-correlated mRNAs are beneficial for us to further investigate the potential mechanism of miRNAs in the development of NPC radioresistance. Herein, mRNAs in “human T-cell leukaemia virus (HTLV) -1 infection pathway” (with most up-regulated genes involved) and “Pathways in cancers” (with most down-regulated genes involved) were detected in both CNE-2-Rs and 6-10B-Rs. Our results demonstrated that 5 of the 6 up-regulated mRNAs in “HTLV-1 infection pathway” increased (*ICAM1*, *WNT2B*, *MYC*, *HLA-F* and TGF-β1) and 3 of the 5 down-regulated mRNAs in “Pathways in cancers” decreased (*CDH1*, *PTENP1*, *HSP90AA1*) (Figure 5). However, there are 3 mRNAs (*HLA-B, FOS* and *JUN*) with inconsistent alterations between CNE-2-Rs and 6-10-Rs cells

## Discussion

In an effort to better understand the mechanisms underlying radioresistance, different radioresistant cell models, including the glioma cell line MGR2R [30], the glioblastoma cell line U251 [31], the colon adenocarcinoma cell line WiDr [32] and the small lung cancer cell line H69 [33], have been generated using distinct IR exposure methods. Repeated low-dose IR exposure and sub-lethal IR exposure were the most frequently adopted strategies that were used to establish radioresistant cancer cell models. In NPC, poorly differentiated cell lines have been the preferred choice for the generation of radioresistant cell models because the majority of NPCs are categorized as poorly differentiated squamous cell carcinomas.

Recently, different radioresistant CNE-2 cell models have been established via sub-lethal IR exposure [4] or repeated low-dose IR exposure [5,16]. In the present study, the radioresistant CNE-2 cell model was successfully established using gradually increasing doses of IR exposure (duration of 5-6 months and a total dose of 60 Gy IR exposure) and multiple validation assays were used to confirm its radioresistant phenotype. Subsequently, another radioresistant 6-10B cell model was also successfully established and validated via the same way. In our opinion, the gradually increasing doses of IR exposure decreased the potential for cellular contamination after repeated low-dose IR exposure and avoided sudden cell death during sub-lethal IR exposure. However, the manner in which IR exposure can maximally enhance the radioresistant capability remains to be investigated in future.

miRNAs are considered to be involved in multiple malignant cell behaviors including radioresistance. The identification of miRNAs that are associated with radioresistance may lead to more individualized and efficient treatments for NPC patients. Previous studies have used miRNA arrays to establish different miRNA expression profiles that are associated with cancer initiation and progression. In the present study, high-throughput deep sequencing technology was applied to both identify known miRNAs associated with NPC radioresistance and discover novel miRNAs associated with NPC radioresistance. Our results demonstrated that 14 known miRNAs were down-regulated and 36 known miRNAs were up-regulated. Some of the previously identified miRNAs, such as miR-125a-3p [34,35], miR-149-3p [36–38], miR-375 [39-41] and miR-508-3p [42], have been reported to regulate the proliferation, apoptosis, migration and invasion of cancer cells and have prognostic value. However, none of these miRNAs has been investigated in cancer radioresistance. Based on a literature review, miR-149-3p [36–38], miR-34c-5p [43,44], miR-375 [39-41] and miR-139-3p [45,46] are inconsistently expressed in different tumor tissues. These discrepancies in miRNA expression in diverse cancers may reflect the complexity of miRNA regulation, which further regulates gene expression in cells. It is possible that miRNAs perform context-specific functions depending on the microenvironment of the tumor cells and the type of cancer [16].

Compared to conventional miRNA array platforms, the major advantage of deep sequencing technology is the ability to conduct massive parallel analyses of the genome-wide expression of miRNAs (miRNome), quantification of the expression levels of individual miRNAs (absolute abundance), identification of miRNA sequence variations and the discovery of novel miRNAs [22]. In the current study, 9 novel, aberrantly expressed, radioresistance-associated miRNAs were identified in NPC, which enriched the list of human miRNAs. Finally, a total of 12 known miRNAs and 2 novel miRNAs were detected in radioresistant NPC cells and their parental radiosensitive cells. The expression trends of 6 known miRNAs and 2 novel miRNAs were altered in the predicted manners, although the actual fold change data did not perfectly match the prediction values. The altered expression of miR-324-5p did not differ significantly between radioresistant NPC cells and their parental radiosensitive cells, which may be attributed to the filtering formula which “allowed for up to 4 base mismatches”. Simultaneously, complete different expression of miR-1246, miR-125a, miR-508-3p, miR-509-3-5p, miR-149-3p was observed in the 2 pairs of radioresistant NPC cell models, which can be rationally explained by the reason that our differential miRNA expression profile was obtained from one single radioresistant cell model. The above identified and novel miRNAs are worthy of further investigation to interpret their functions in NPC radioresistance both *in vitro* and *in vivo*.

miRNAs can function as onco-miRNAs or anti-onco-miRNAs depending on their potential target mRNA genes [47]. However, the interactions between an miRNA and its target mRNAs are very complicated. Generally, a biological pathway will involve a set of specific miRNAs and a specific miRNA will target multiple mRNAs. Bioinformatic algorithms have played a critical role in the discovery of miRNAs and the prediction of their target mRNAs. Here, RNAhybrid is a tool that is primarily used to predict miRNA targets [22]. However, thousands of target genes were predicted in the study, of which only a small fraction of the target genes actually participated in the process of NPC radioresistance. Therefore, a series of reduction strategies were applied to reduce the target mRNAs including construction of radioresistance associated mRNAs expression profile and filtering out the remained genes (intersection of predicted mRNAs and differential mRNA expression profile) with the nasopharyngeal- and NPC-specific genes. Finally, the target list was narrowed down to limited 53 mRNAs, including 33 up-regulated and 20 down-regulated mRNAs.

To more thoroughly understand the function of miRNAs, KEGG analyses futher indicated that the predicted 53 target mRNAs were involved in 37 signaling pathways. Direct and indirect evidence revealed the critical roles that the TGF-beta, WNT and Toll-like receptor signaling pathway played in the process of radioresistance [48-50]. Interestingly, other signaling pathways including bladder cancer pathway, thyroid cancer pathway and colorectal cancer pathway were also included, which implicated that specific signaling pathways functioned in malignant behaviours in a broad spectrum of human cancers [51,52]. In addition, viral infection is a common cause of oncogenesis and may provide clues that will help us study this EBV-related tumor and its potential connection to radioresistance [53,54]. Several pathways did not seem to be directly connected to radioresistance or cancer at first glance. This may be attributed to the fact that the KEGG analyses were based on the data that were currently available online and that the genes involved in these pathways were annotated not only to cancer or radioresistance, but also to other diseases or biological functions. 

Finally, partial pathways-correlated target mRNAs were validated in this study. Our data showed that 8 mRNAs had corresponding changes as predicted in these two radioreisitant cell models. Among the 8 mRNAs，*WNT2B* has been reported to participate in the miR-324-3p mediated NPC radioreisitance in our previous publication [13]. *TGF-β1* is an endogenous radioresistance factor in the esophageal adenocarcinoma [55] and the expression of *ICAM1* is affected by IR [56]. *CDH1* enhances the radiosensitivity in NPC cells via mediating the epithelial-mesenchymal transition (EMT) occurrence [57]. All the above reports have suggested that some validated mRNAs play important role in the regulation of radioresistance, but the validated mRNAs that are not reported and the remain unvalidated mRNAs need to be investigated for their potential roles in radioresistance in future. Importantly, this predicted targets is helpful for the functional and mechanismistic studies in the radioresistance associated miRNAs.

Taken together, the results of the present study offer a solid foundation for the deeper investigation of miRNA regulatory networks in NPC radioresistance. The results of the in-depth sequencing and analysis of radioresistance associated miRNAs in NPC that was presented here will provide a solid foundation for the future exploration of their potential roles in the regulation of radioresistance.

## Supporting Information

Table S1 (XLS)

Table S2 (XLS)

Table S3 (XLS)

Figure S1 **Radioresistant 6-10B cells with radioresistance (6-10B-Rs) cells are established and validated.** (A) The survival rates for 6-10B-Rs and 6-10B cells at different irradiation (IR) doses (2, 4, 6 and 8 Gy) and at different time points (1, 2, 3, 4 and 5 days) were determined using a CCK-8 assay. (B) The growth curves of 6-10B-Rs and 6-10B cells exposed or not exposed to 4 Gy IR. (C) A representative image of colony formation in 6-10B-Rs and 6-10B cells exposed to or not exposed to different doses of IR after 14 days (Left). Survival fractions of 6-10B-Rs and 6-10B cells were obtained from the results of the colony-forming assays. (D) Apoptotic changes in 6-10B-Rs and 6-10B cells exposed or not exposed to 4 Gy IR for 72 h. The results were the average of three independent experiments ± standard deviation (S.D) (*p < 0.05; **p < 0.01).
(TIF)
