# Correction: Genome-Wide Analyses of Radioresistance-Associated miRNA Expression Profile in Nasopharyngeal Carcinoma Using Next Generation Deep Sequencing

**DOI:** 10.1371/journal.pone.0110099

**Published:** 2014-10-01

**Authors:** The PLOS ONE Staff

In the Funding section, two grant numbers from the funder National Natural Science Foundation of China are missing. The correct grant numbers: No.81372426, No.81372906, No.81202128 and No.81272974.
