# Supplementary figures and images for: Genome-Wide Analyses of Radioresistance-Associated miRNA Expression Profile in Nasopharyngeal Carcinoma Using Next Generation Deep Sequencing
Source: PLoS One. 2013 Dec 19;8(12):e84486. doi: 10.1371/journal.pone.0084486 (PMC3868612; doi:10.1371/journal.pone.0084486)

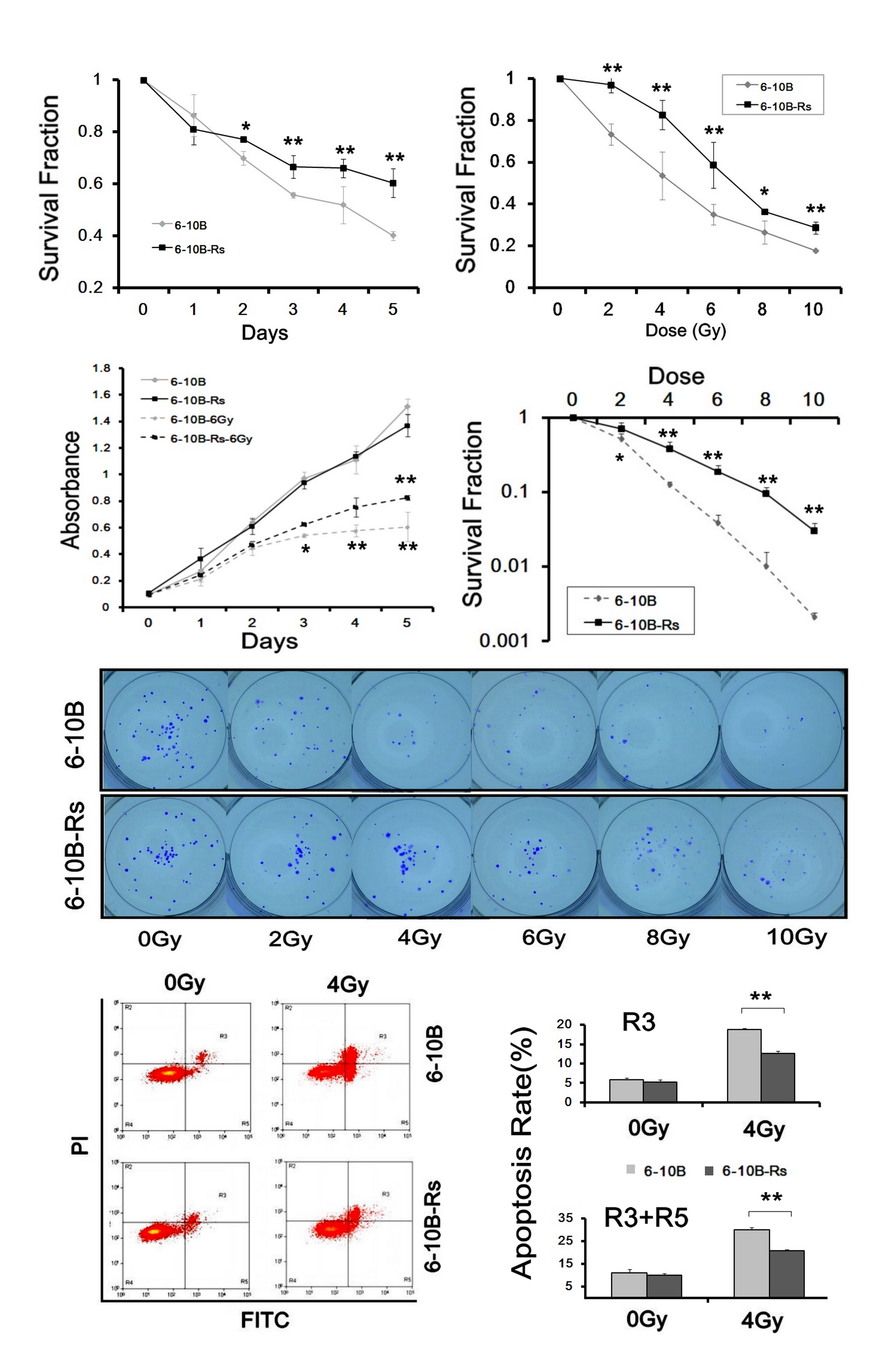

Supplement: Figure S1 — Radioresistant 6-10B cells with radioresistance (6-10B-Rs) cells are established and validated. (A) The survival rates for 6-10B-Rs and 6-10B cells at different irradiation (IR) doses (2, 4, 6 and 8 Gy) and at different time points (1, 2, 3, 4 and 5 days) were determined using a CCK-8 assay. (B) The growth curves of 6-10B-Rs and 6-10B cells exposed or not exposed to 4 Gy IR. (C) A representative image of colony formation in 6-10B-Rs and 6-10B cells exposed to or not exposed to different doses of IR after 14 days (Left). Survival fractions of 6-10B-Rs and 6-10B cells were obtained from the results of the colony-forming assays. (D) Apoptotic changes in 6-10B-Rs and 6-10B cells exposed or not exposed to 4 Gy IR for 72 h. The results were the average of three independent experiments ± standard deviation (S.D) (*p < 0.05; **p < 0.01). (TIF) [file pone.0084486.s004.tif]
